# Newly identified genes contribute to vanillin tolerance in *Saccharomyces cerevisiae*

**DOI:** 10.1111/1751-7915.13643

**Published:** 2020-07-30

**Authors:** Zhenzhen Liang, Xinning Wang, Xiaoming Bao, Tiandi Wei, Jin Hou, Weifeng Liu, Yu Shen

**Affiliations:** ^1^ State Key Laboratory of Microbial Technology Institute of Microbial Technology Shandong University Qingdao 266237 China; ^2^ State Key Laboratory of Biobased Material and Green Papermaking School of Bioengineering Qi Lu University of Technology Jinan 250353 China

## Abstract

Exploring the mechanisms of tolerance in microorganisms to vanillin, which is derived from lignin, will benefit the design of robust cell factories that produce biofuels and chemicals using lignocellulosic materials. Our objective was to identify the genes related to vanillin tolerance in *Saccharomyces cerevisiae*. We investigated the effects on vanillin tolerance of several genes that have site mutations in the highly vanillin‐tolerant strain EMV‐8 compared to its parental line NAN‐27. The results showed that overexpression of *GCY1*, a gene that encodes an aldo‐keto reductase that also has mRNA‐binding activity*, YPR1*, a paralog of *GCY1* that encodes an aldo‐keto reductase*, PEX5*, a gene that encodes a peroxisomal membrane signal receptor and *MBF1*, a gene that encodes a multiprotein bridging factor increase the specific growth rates (μ) by 49%, 41%, 44% and 48 %, respectively, in medium containing 6 mmol l^−1^ vanillin. Among these gene products, Gcy1p and Ypr1p showed NADPH‐dependent and NAD(P)H‐dependent vanillin reductase activity, respectively. The reductase‐inactive mutant Gcy1p^Y56F^ also increased vanillin tolerance in *S. cerevisiae*, suggesting that other mechanisms exist. Although *TRS85* and *PEX5*, genes for which the mRNAs are binding targets of Gcy1p, were shown to be related to vanillin tolerance, both the mRNA and protein levels of these genes were not changed by overexpression of *GCY1*. The relationship between the mRNA‐binding activity of Gcy1p and its positive effect on vanillin tolerance is still not clear. Finally, we found that the point mutation D112A in Mbf1p, which disrupts the binding of Mbf1p and the TATA element‐binding protein (TBP), did not decrease the positive effect of Mbf1p on vanillin tolerance. This indicates that the binding of Mbf1p and TBP is not necessary for the positive effect on vanillin tolerance mediated by Mbf1p. We have successfully identified new genes related to vanillin tolerance and provided novel targets that can be used to improve the vanillin tolerance of *S. cerevisiae*. Moreover, we have extended our understanding of the proteins encoded by these genes.

## Graphical abstract

We identified that overexpression of *GCY1*, *YPR1*, *PEX5*, and *MBF1* enhance the vanillin tolerance of *Saccharomyces cerevisiae* and explored some of the related mechanisms. This provided novel targets that can be used to improve the performance of *S. cerevisiae* used in 2nd generation fuel ethanol production, and extended our understanding of the proteins encoded by these genes.

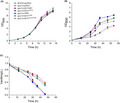

## Introduction

Bioconversion of lignocellulosic materials, which are readily available and renewable, for production of biofuel and other valuable products has been the subject of intense studies (Balat, 2011; Koutinas *et al*., 2016; Bilal *et al*., 2017, Ramos and Duque, 2019). Pre‐treatment is an unavoidable process that is necessary to break the crosslinking of cellulose, hemicellulose, and lignin in lignocellulosic biomass, and to then facilitate the enzymatic hydrolysis of cellulose and the release of monosaccharides (Alvarez *et al*., 2016, Ramos and Duque, 2019). However, a range of harmful chemicals are generated during this process (Balat, 2011; Sindhu *et al*., 2016). Vanillin, a low‐molecular‐mass guaiacyl phenol, is an important toxic by‐product of pre‐treated lignocellulosic biomass. Both the types of biomass materials and the pre‐treatment conditions affect the concentration of vanillin, which ranges between 1 and 26 mM (Almeida *et al*., 2007, Heer and Sauer 2008). However, vanillin inhibits cell viability in many microorganisms, such as *Escherichia coli* and *Saccharomyces cerevisiae*, at very low concentrations (Klinke *et al*., 2004). Moreover, vanillin is one of the most widely used flavouring agents in the food industry. Efforts have been made to produce vanillin by microorganisms from other components of lignin, such as ferulic acid (Li and Zheng 2020, Liang *et al*., 2020), and some efforts have been directed towards de novo synthesis of vanillin from glucose (Hansen *et al*., 2009). Exploring the cellular mechanisms related to vanillin tolerance is important for constructing robust cellular factories that either utilize the lignocellulosic materials or that produce vanillin (Wang *et al*., 2017, Pattrick et al. 2019).

*Saccharomyces cerevisiae* is considered to be the most competitive microorganism for biofuel production due to better fermentative performance, high tolerance to ethanol and ease of genetic manipulation (Liu *et al*., 2009; Zhang *et al*., 2010). It is also considered to be a potential vanillin producing cell factory (Hansen *et al*., 2009). The effects of vanillin on *S. cerevisiae* are revealing: (i) vanillin disrupts the integrity of biological membranes which causes disequilibrium in the intra‐ and extracellular ions (Fitzgerald *et al*., 2004); (ii) vanillin represses translation by affecting the function of the large ribosomal subunit and leads to the formation of processing bodies and stress granules (Iwaki *et al*., 2013; Ishida *et al*., 2016); and (iii) vanillin induces oxidative stress and mitochondrial fragmentation (Nguyen *et al*., 2014).

Correspondingly, maintaining the fluidity and stability of the membrane by increasing the ergosterol content and enhancing ribosome biogenesis are advantageous to strains resistant to vanillin (Endo *et al*., 2008; Endo *et al*., 2009; Wang *et al*., 2017). Enhancing the antioxidative response and glutathione biosynthesis are beneficial to strains that are resistant to lignocellulose‐derived inhibitors (Kim *et al*., 2006; Ask *et al*., 2013; Kim *et al*., 2014). Increasing the ability of yeast strains to convert an aldehyde group, which is responsible for the toxicity of vanillin, to a hydroxyl group is an effective way to improve vanillin tolerance in yeast (Larroy *et al*., 2002; Liu, 2011; Shen *et al*., 2014; Wang *et al*., 2016).

Despite the fact that there is increasing interest in understanding vanillin tolerance in yeast, there are few previous studies that focused on the underlying molecular mechanisms. More vanillin reductases and genes in the antioxidative response systems involved in vanillin tolerance need to be identified, and the correlation between the DNA repair response and vanillin tolerance should be studied. In our previous work, the genome sequence of the vanillin‐tolerant strain EMV‐8 was compared with that of its parent strain, NAN‐27, and > 450 non‐synonymous SNPs and 44 InDels were identified (Wang *et al*., 2017). We then studied the effects of all the InDels and found that the deletion of a transcription factor gene, *YRR1*, significantly increased vanillin tolerance in *S. cerevisiae* (Wang *et al*., 2017).

In this study, we have continued to examine the relationship between vanillin tolerance and the non‐synonymous SNPs we identified in the genome of EMV‐8. Specifically, we focused on SNPs in the genes that encode oxidoreductases and the genes that respond to oxidative stress or DNA repair. We found that overexpression of *GCY1* and *MBF1* enhanced vanillin tolerance in yeast strain BY4741. We then demonstrated that Gcy1p has NADPH‐dependent vanillin reductase activity, which can directly increase vanillin tolerance. However, the Gcy1p mutant with abolished vanillin reductase activity still showed enhanced vanillin tolerance. Moreover, overexpression of *TRS85* and *PEX5*, genes in which the mRNAs are the binding targets of Gcy1p, weakened and improved vanillin tolerance, respectively. However, neither the mRNA nor the protein levels of *TRS85* and *PEX5* changed as a result of overexpressing *GCY1*. Finally, we demonstrated that weakening the interaction between Mbf1p and TBP did not affect the positive effect of Mbf1p on vanillin tolerance, which suggests that Mbf1p enhances vanillin tolerance independent of its TBP binding activity.

## Results and discussion

### Selection of SNPs that are potentially related to vanillin tolerance in *S. cerevisiae*

**Fig. 1:**
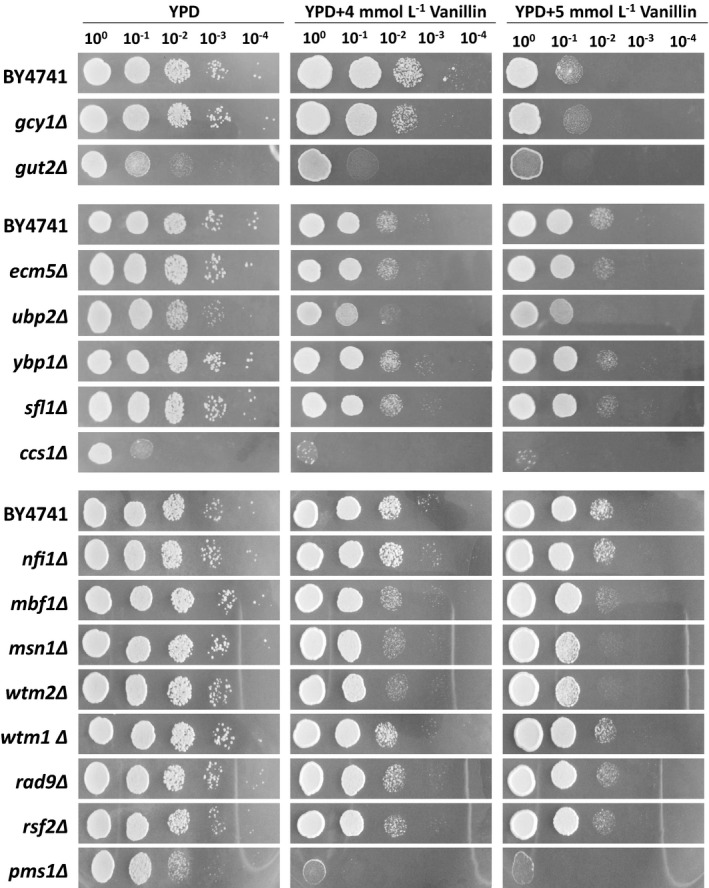
Growth profiles of yeast strains on medium with or without vanillin. Cells cultured overnight were harvested and diluted to an OD_600_ of 1.0. Ten‐fold serial dilutions were then spotted onto plates containing YPD medium, YPD containing 4 mmol l^−1^ vanillin, and YPD containing 5 mmol l^−1^ vanillin, and incubated at 30°C for 1, 2 and 3 days, respectively.

In our previous work, >450 non‐synonymous SNPs were identified between the genome sequences of the vanillin‐tolerant strain EMV‐8 and its parent strain NAN‐27 (Wang *et al*., 2017). Here, the SNPs in the genes that encode oxidoreductases and those in genes that respond to oxidative stress or DNA repair were selected, because vanillin damages *S. cerevisiae* in these aspects. The gene sequences were first amplified from the genomes of EMV‐8 and NAN‐27, and the PCR fragments were sequenced to confirm that the mutations were consistent with the genomic defects. The confirmed genes (Table 1) were then deleted in *S. cerevisiae*, and the resulting mutant strains were spotted onto YPD plates with or without vanillin to determine their effect on vanillin tolerance. The results revealed that deletion of *GCY1*, *MBF1*, *MSN1* or *WTM2* did not affect the growth of strains on YPD medium but retarded the growth of the strains on YPD containing vanillin (Fig. 1). This suggests that deleting these genes makes yeast more sensitive to vanillin, and these genes may relate to vanillin tolerance.

**Table 1:** Non‐synonymous SNPs in the oxidoreductase genes and the genes that respond to oxidative stress and DNA repair in EMV‐8 compared with NAN‐27.

Genes	Function	Mutation(s) in proteins (EMV‐8 vs. NAN‐27)
*Genes Encoding Oxidoreductase*		
*GCY1*	Putative NADP^+^ coupled glycerol dehydrogenase; member of the aldo‐keto reductase (AKR) family; also has mRNA‐binding activity.	A181E
*GUT2*	Glycerol‐3‐phosphate dehydrogenase.	R8K
*Genes Related to Oxidative Stress Response*		
*ECM5*	Subunit of the Snt2C complex; oxidative stress response protein.	R54G
*UBP2*	Ubiquitin‐specific protease.	S282N
*YBP1*	oxidative stress response protein.	T12A
*SFL1*	Transcriptional repressor and activator; activator of stress responsive genes.	W453S/Y497F
*CCS1*	Copper chaperone for superoxide dismutase Sod1p; oxidative stress protection protein.	Q94R
*Genes Related to DNA Repair*		
*NFI1*	Regulator of telomerase activity; multisite modificator of several DNA repair proteins.	R345K
*MBF1*	Transcriptional coactivator that bridges the DNA‐binding region of Gcn4p and TATA‐binding protein Spt15p; suppressor of frameshift mutations.	P148L
*MSN1*	Transcriptional activator involved in regulation of invertase and glucoamylase expression, invasive growth and pseudohyphal differentiation, iron uptake, chromium accumulation and response to osmotic stress.	S171P/P190Q^a^
*WTM2*	Transcriptional modulator involved in regulation of meiosis, silencing, and expression of RNR genes; involved in response to replication stress.	Q8L
*WTM1*	Transcriptional modulator involved in regulation of meiosis, silencing and expression of RNR genes; required for nuclear localization of the ribonucleotide reductase small subunit Rnr2p and Rnr4p.	A71S
*RAD9*	DNA damage‐dependent checkpoint protein.	I1296M
*RSF2*	Zinc‐finger protein involved in transcriptional control of both nuclear and mitochondrial genes.	V79A
*PMS1*	ATP‐binding protein required for mismatch repair, mitosis and meiosis.	N41S^a^/I112T/ A401S/R822K^a^

^a^ The results of the large‐scale sequencing were verified/revised by re‐sequencing the PCR‐amplified gene fragments.

### Overexpression of *GCY1* increases vanillin tolerance in *S. cerevisiae*

**Fig. 2:**
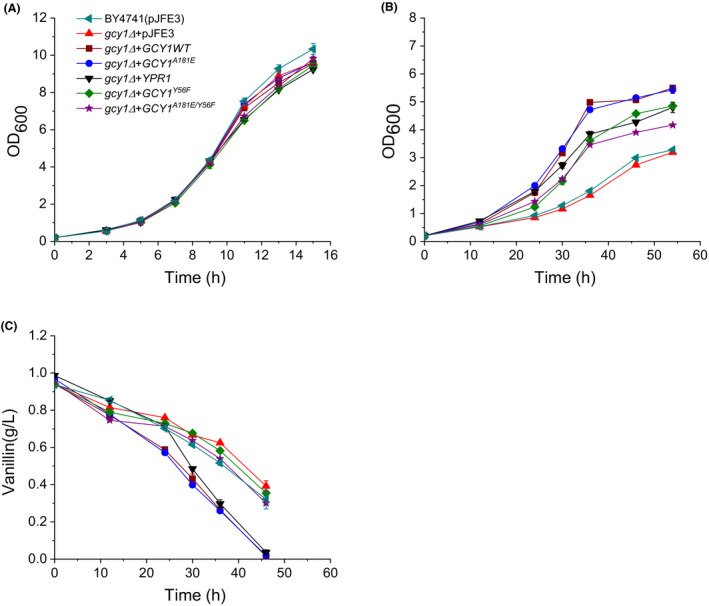
Growth and vanillin reduction curves of recombinant *S. cerevisiae* strains carrying *GCY1* alleles in medium with or without vanillin. (A) growth curve in SC‐ura medium; (B) growth curve in SC‐ura medium containing 6 mmol l^−1^ vanillin; (C) vanillin reduction in SC‐ura medium containing 6 mmol l^−1^ vanillin. The cells were cultured in 40 ml SC‐ura or SC‐ura + 6 mmol l^−1^ vanillin media at 30°C with shaking (200 rpm) starting with an initial OD_600_ of 0.2. Data are the mean values of triplicate tests. Symbols: 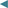, BY4741(pJFE3); 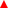, BY4741(*gcy1Δ + pJFE3*); 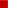, BY4741(*gcy1Δ + GCY1^WT^*); 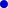, BY4741(*gcy1Δ + GCY1^A181E^*); ▼, BY4741(*YPR1*); 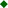, BY4741(*gcy1Δ + GCY1^Y56F^*); 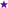, BY4741(*gcy1Δ + GCY1^A181E/Y56F^*).

To investigate the role of *GCY1* and the mutant A181E in vanillin tolerance in *S. cerevisiae*, the *GCY1^WT^* and *GCY1^A181E^* alleles were cloned from the genomic DNA of strains NAN‐27 and EMV‐8, respectively, and were individually overexpressed in strain BY4741(*gcy1Δ*). BY4741 transformed with the empty vector pJFE3, designated BY4741(pJFE3), was used as the control. The vanillin tolerance of the strains was then evaluated. In vanillin‐free SC‐ura medium, all strains showed similar specific growth rates (μ) during the exponential growth phase. However, compared with the control BY4741(pJFE3), the μ of BY4741(*gcy1Δ + GCY1^WT^*) and BY4741(*gcy1Δ + GCY1^A181E^*) increased by 49% (*P*‐value < 0.01) and 54% (*P*‐value < 0.01), respectively, in the presence of 6 mmol l^−1^ vanillin. Moreover, the specific vanillin reduction rates (*r*_vanillin_) of BY4741(*gcy1Δ + GCY1^WT^*) and BY4741(*gcy1Δ + GCY1^A181E^*) were 36% (*P*‐value < 0.01) and 34% (*P*‐value < 0.01) higher than in the BY4741(pJFE3) control, respectively (Fig. 2 and Table 2). Also, there was no significant difference between strains BY4741(*gcy1Δ + GCY1^WT^*) and BY4741(*gcy1Δ + GCY1^A181E^*). These results show that overexpression of *GCY1* improved the vanillin tolerance of yeast strain BY4741 and that the point mutation A181E did not further improve vanillin tolerance.

**Table 2:** Growth and vanillin reduction parameters of recombinant *S. cerevisiae* strains in SC‐ura medium with or without vanillin.

Strains	SC‐ura medium	SC‐ura medium with 6 mmol l^−1^ vanillin	
	µ (h^−1^)	µ (h^−1^)	*r*_vanillin_ (g l^−1^ h^−1^ g^−1^ DCW)
BY4741(pJFE3)	0.345 ± 0.002	0.061 ± 0.000	0.044 ± 0.005
BY4741(*gcy1Δ + pJFE3*)	0.341 ± 0.002	0.057 ± 0.000	0.047 ± 0.005
BY4741(*gcy1Δ + GCY1^WT^*)	0.342 ± 0.004	0.091 ± 0.000*	0.060 ± 0.008*
BY4741(*gcy1Δ + GCY1^A181E^*)	0.344 ± 0.000	0.094 ± 0.000*	0.059 ± 0.002*
BY4741(*YPR1*)	0.345 ± 0.001	0.086 ± 0.001*	0.062 ± 0.001*
BY4741(*gcy1Δ + GCY1^Y56F^*)	0.334 ± 0.004	0.077 ± 0.002*	0.045 ± 0.003
BY4741(*gcy1Δ + GCY1^A181E/Y56F^*)	0.340 ± 0.002	0.080 ± 0.000*	0.045 ± 0.002
BY4741(*mbf1Δ + pJFE3*)	0.383 ± 0.007	0.051 ± 0.001	0.046 ± 0.006
BY4741(*mbf1Δ + MBF1^WT^*)	0.353 ± 0.000	0.090 ± 0.002*	0.047 ± 0.001
BY4741(*mbf1Δ + MBF1^P148L^*)	0.352 ± 0.000	0.088 ± 0.001*	0.047 ± 0.001
BY4741(*mbf1Δ + MBF1^D112A^*)	0.323 ± 0.003	0.092 ± 0.001*	0.049 ± 0.001
BY4741(*mbf1Δ + MBF1^P148L/D112A^*)	0.328 ± 0.000	0.091 ± 0.001*	0.049 ± 0.001

μ, the specific growth rates; DCW, dry cell weight; *r*_vanillin_, the specific vanillin reduction rate.

The parameters were calculated basing on the data of exponential growth phase and are given as the averages ± standard deviation of the biological triplicates.

* *P*‐value < 0.01.

### The NADPH‐dependent vanillin reductase activity of Gcy1p contributes to vanillin tolerance in *S. cerevisiae*

**Fig. 3:**
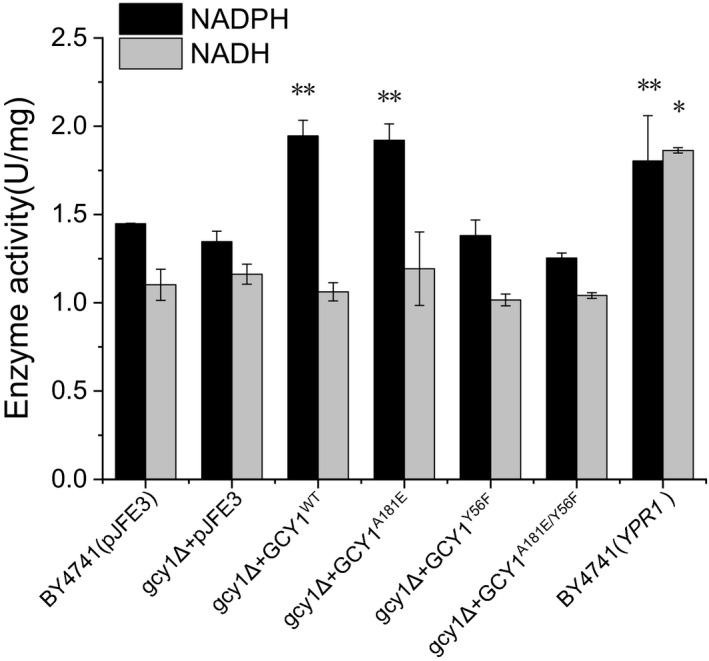
Vanillin reductase activity in cell‐free extracts of recombinant yeast strains. Data are mean values ± standard deviations of three replicates. *P‐value < 0.1, **P‐value < 0.01.

**Fig. 4:**
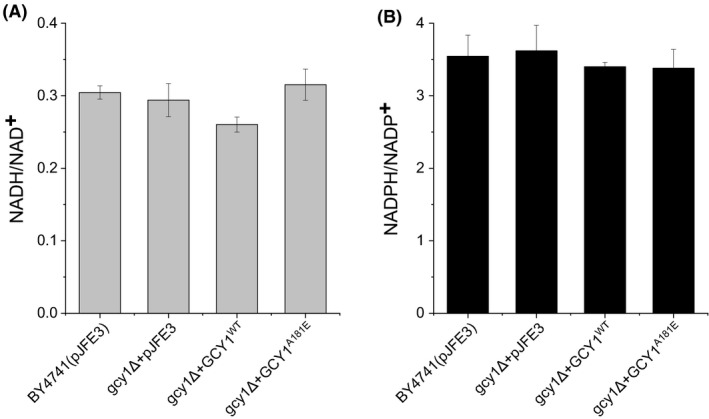
The NADH/NAD^+^ and NAD(P)H/NAD(P)^+^ ratios in recombinant *S. cerevisiae* strains. Data are mean values ± standard deviations of three replicates.

It is generally considered that the capacity of a yeast strain to convert vanillin to vanillyl alcohol and the quantity of the reduced coenzyme that can be used are strongly correlated with the strain’s level of vanillin tolerance (Liu, 2011; Wang *et al*., 2016). Furthermore, overexpression of Gcy1p, which is a member of the aldo‐keto reductase (AKR) family, increased the *r*_vanillin_ of the strain as mentioned above. We next measured the vanillin reductase activity of the yeast strains. Results of the enzyme assays showed that the vanillin reductase activity with NADPH as a cofactor was increased by 34% (*P*‐value < 0.01) and 33% (*P*‐value < 0.01) in BY4741(*gcy1Δ + GCY1^WT^*) and BY4741(*gcy1Δ + GCY1^A181E^*), respectively, compared to the BY4741(pJFE3) control. However, the vanillin reductase activities measured in these strains in the presence of NADH are not significantly different (Fig. 3). This suggests that Gcy1p has NADPH‐dependent but not NADH‐dependent vanillin reductase activity. We then determined the ratio of NAD(P)H to NAD(P)^+^ in the strains. The results showed that the ratios of NAD(P)H to NADP^+^ in the strains BY4741(*gcy1Δ + GCY1^WT^*) and BY4741(*gcy1Δ + GCY1^A181E^*) were similar to that of the control BY4741(pJFE3) (Fig. 4). These results indicate that overexpression of *GCY1* does not affect [NAD(P)H]/[NAD(P)^+^]. Taken together, our results suggest that Gcy1p enhances vanillin tolerance in *S. cerevisiae* not by increasing the supply of reduced coenzyme, but rather by directly reducing vanillin, usually to form less toxic vanillyl alcohol (Wang *et al*., 2016).

### Ypr1p, the paralog of Gcy1p, has NAD(P)H‐dependent vanillin reductase activity and increases vanillin tolerance in *S. cerevisiae*

Gcy1p has a paralog, Ypr1p, with which it shares approximately 65% sequence homology (Chang *et al*., 2007). We also evaluated the vanillin tolerance and determined the vanillin reductase activity of a strain overexpressing *YPR1*. The results showed that the *YPR1*‐overexpressing strain, BY4741(*YPR1*), grew as well as the control BY4741(pJFE3) in vanillin‐free medium, but it grew markedly faster than the control, and the *r*_vanillin_ increased by 41% (*P*‐value < 0.01) compared to that of BY4741(pJFE3), in the presence of 6 mmol l^−1^ vanillin (Fig. 2 and Table 2). Furthermore, enzyme assay results showed that the crude extracts of BY4741(*YPR1*) had 25% (*P*‐value < 0.1) higher NADPH‐dependent vanillin reductase activity and 69% (*P*‐value < 0.01) higher NADH‐dependent catalytic activity compared with BY4741(pJFE3) (Fig. 3). These results show that Ypr1p has both NADH‐ and NADPH‐dependent vanillin reductase activity and that overexpression of *YPR1* increased vanillin tolerance in yeast.

An important and efficient way to detoxify vanillin is to convert it to the less toxic vanillyl alcohol. In previous reports, Gcy1p has been shown to exhibit high catalytic efficiencies towards most substrates with carbonyl and aldehyde groups, especially benzaldehydes and phenylglyoxal (Magdolen *et al*., 1990; Chang *et al*., 2007). Ypr1p has a high specific activity towards 2‐methylbutyraldehyde (Ford and Ellis 2002, Chang *et al*., 2007). Our results confirmed that Gcy1p and Ypr1p also have vanillin reductase activity, as do the previously reported Adh6p and Adh7p proteins and the proteins encoded by *YNL134C* and *YJR096W* (Larroy *et al*., 2002; Nguyen *et al*., 2015; Wang *et al*., 2016).

### Overexpression of Gcy1p^Y56F^, the vanillin reductase‐inactive mutant allele, also increases vanillin tolerance in *S. cerevisiae*

It has been reported that the tyrosine‐56 of yeast Gcy1p is the presumptive proton donor in aldehyde reduction (Tarle *et al*., 1993, Hur and Wilson 2001) and that mutation of Y56F dramatically reduced the catalytic efficiency of this enzyme to reduce DL‐glyceraldehyde (Chang *et al*., 2007). To determine whether the reduction of vanillin is the only reason that overexpression of *GCY1* increases vanillin tolerance in *S. cerevisiae*, we also constructed a strain overexpressing Gcy1p^Y56F^, the reductase‐inactive mutant of Gcy1p, and evaluated its effect on vanillin tolerance.

The vanillin reductase activities of crude extracts of the mutant yeast strains were assayed. The results showed that with NADPH as a cofactor, the vanillin reductase activities of BY4741(*gcy1Δ + GCY1^Y56F^*) and BY4741(*gcy1Δ + GCY1^A181E/Y56F^*) were decreased by 29% and 35%, respectively, compared to BY4741(*gcy1Δ + GCY1^WT^*) and BY4741(*gcy1Δ + GCY1^A181E^*) and were similar to that of the BY4741(pJFE3) control (Fig. 3). Corresponding to the low vanillin reductase activities, the *r*_vanillin_ of BY4741(*gcy1Δ + GCY1^Y56F^*) and BY4741(*gcy1Δ + GCY1^A181E/Y56F^*) was reduced by 25% and 24%, respectively, compared to BY4741(*gcy1Δ + GCY1^WT^*) and BY4741(*gcy1Δ + GCY1^A181E^*), and the values were similar to that of BY4741(pJFE3), in medium containing 6 mmol l^−1^ vanillin. Moreover, the growth rates (μ) of BY4741(*gcy1Δ + GCY1^Y56F^*) and BY4741(*gcy1Δ + GCY1^A181E/Y56F^*) were both decreased compared to BY4741(*gcy1Δ + GCY1^WT^*) in medium containing 6 mmol l^−1^ vanillin. Unexpectedly however, the μ of BY4741(*gcy1Δ + GCY1^Y56F^*) and BY4741(*gcy1Δ + GCY1^A181E/Y56F^*) were still 26% (*P*‐value < 0.01) and 31% (*P*‐value < 0.01), higher, respectively, than that of BY4741(pJFE3) (Fig. 2 and Table 2). These results suggest that the Y56F mutation abolished the vanillin reductase activity of Gcy1p, but overexpressing the catalytically‐inactive Gcy1p^Y56F^ still increased vanillin tolerance in the yeast strains. Thus, there are reasons that overexpressing Gcy1p increased vanillin tolerance other than its vanillin reductase activity.

### The mRNA‐binding activity of Gcy1p does not affect the mRNA and protein levels of *TRS85* and *PEX5*, which have a positive effect on vanillin tolerance in yeast

**Fig. 5:**
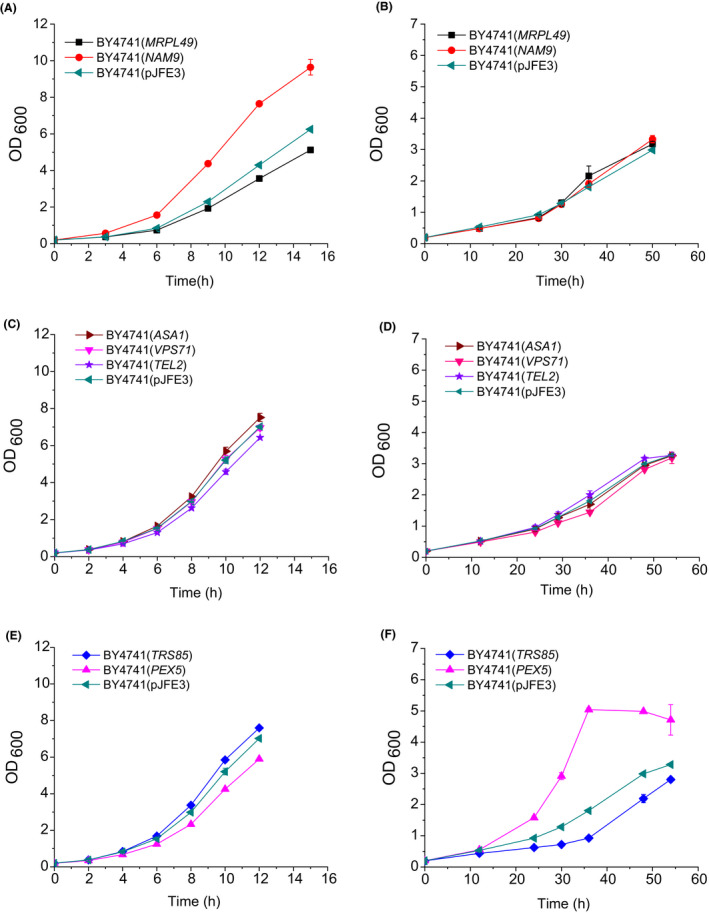
Growth kinetics of recombinant yeast strains in SC‐ura medium (A, C, and E) and in SC‐ura medium containing 6 mmol l^−1^ vanillin (B, D, and F). The cells were cultured in 40 ml SC‐ura or SC‐ura + 6 mmol l^−1^ vanillin at 30°C with shaking (200 rpm) starting with an initial OD_600_ of 0.2. Data are the mean values of triplicate tests. Symbols: 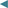, BY4741(pJFE3); ■, BY4741(*MRPL49*); 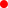, BY4741(*NAM9*); 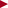, BY4741(*ASA1*); 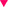, BY4741(*VPS71*); 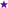, BY4741(*TEL2*); 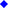, BY4741(*TRS85*); 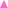, BY4741(*PEX5*).

In addition to being a known member of the aldo‐keto reductase (AKR) family, Gcy1p was also found to be an mRNA‐binding protein (RBP) with broad specificity that can interact with 44 mRNAs (Tsvetanova *et al*., 2010). To determine whether the mRNA‐binding function of Gcy1p is related to vanillin tolerance, mRNAs transcribed from three types of genes were selected, and their effects on vanillin tolerance were evaluated. Specifically, genes related to ribosomal proteins (*MRPL49* and *NAM9*), chromatin organization, (*ASA1*, *VPS71* and *TEL2*) and peroxisomes (*TRS85* and *PEX5*) were cloned from the genomic DNA of strain BY4741 and then overexpressed in the same strain. The growth characteristics of the resulting strains (Fig. 5) showed that overexpression of *MRPL49*, *NAM9*, *ASA1*, *VPS71* and *TEL2* did not affect the μ of the strains cultured in medium containing vanillin. However, the μ of BY4741 (*TRS85*) was 0.042 ± 0.001 (h^−1^), which was 31% (*P*‐value < 0.01) lower than that of the control BY4741(pJFE3). Also, the μ of BY4741(*PEX5*) was 0.088 ± 0.001 (h^−1^), which was 44% (*P*‐value < 0.01) higher than that of BY4741(pJFE3) when the strains were cultured in vanillin‐containing medium. These results show that overexpression of the *TRS85* and *PEX5* genes can affect vanillin tolerance in yeast.

Vanillin induces intracellular ROS accumulation, which causes oxidative damage such as mitochondrial fragmentation (Nguyen *et al*., 2014). Pex5p is a peroxisomal membrane signal receptor for the C‐terminal tripeptide signal sequence (PTS1) of peroxisomal matrix proteins, and it is required for peroxisomal matrix protein import. Among the genes that encode proteins containing the PTS1 sequence (http://www.peroxisomedb.org/, accessed 19 Feb, 2020, listed in Table S2), some are related to protecting cells against oxidative damage, such as *AHP1* and *GTO1*; others are important carbon or nitrogen metabolism genes that supply energy for cell growth. We have reason to assume that helping these PTS1‐containing proteins locate to the peroxisome is one of the mechanisms by which overexpressing *PEX5* enhances vanillin tolerance in yeast.

**Fig. 6:**
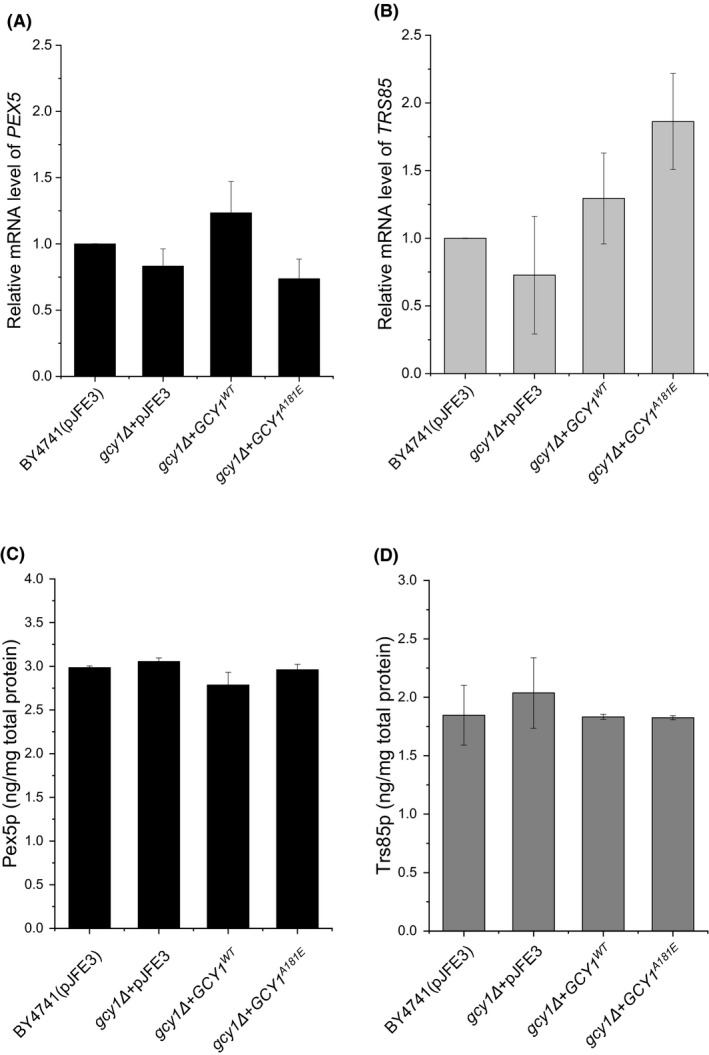
mRNA and protein levels of *PEX5* and *TRS85* in the recombinant yeast strains. (A), relative mRNA levels of *PEX5*; (B), relative mRNA levels of *TRS85*; (C, D) protein levels of Pex5p (C) and Trs85p (D). Each strain had a 6*HIS label fused with the gene sequence in the chromosome. For the sake of simplicity, the 6*His(*in situ*) tag is not shown on the graphs in parts (C) and (D). The data are presented as the means ± standard errors of triplicate.

Although Gcy1p has been identified as an mRNA‐binding protein of *TRS85* and *PEX5*, it is unclear what influence it has on their mRNAs. Therefore, we determined the mRNA and protein levels of both *TRS85* and *PEX5* in yeast strains with various levels of *GCY1* expression. The quantitative PCR results showed that neither deletion nor overexpression of *GCY1* caused significant changes in the mRNA levels of either *TRS85* or *PEX5* (Fig. 6A and B). This suggests that transcription and mRNA stabilization of *TRS85* and *PEX5* are not affected by the level of Gcy1p. Furthermore, the ELISA test results showed that the amounts of Trs85p and Pex5p were similar in all of the strains (Fig. 6C and D), indicating that translation of *TRS85* and *PEX5* mRNAs is not affected by *GCY1* deletion or overexpression. Therefore, overexpression of *GCY^WT^* and *GCY1^A181E^* affected neither the mRNA nor protein levels of *TRS85* and *PEX5*. More effort is required to understand the correlation between Gcy1p, *TRS85*‐ and *PEX5*‐specific mRNAs, and vanillin tolerance in *S. cerevisiae*.

### Overexpression of *MBF1* increases vanillin tolerance in *S. cerevisiae*

**Fig. 7:**
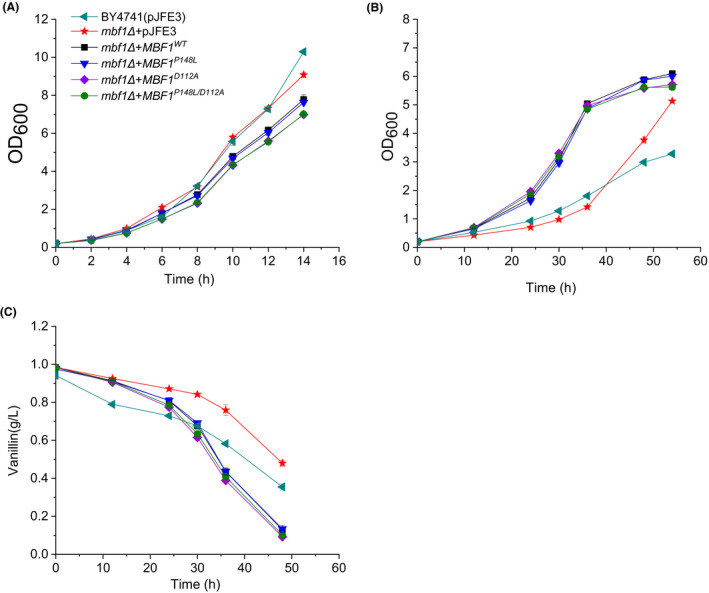
Growth and vanillin reduction curves of recombinant *S. cerevisiae* strains carrying different *MBF1* alleles in medium with or without vanillin. (A) growth curves in SC‐ura medium; (B) growth curves in SC‐ura medium containing 6 mmol l^−1^ vanillin; (C) vanillin reduction in SC‐ura containing 6 mmol l^−1^ vanillin. The cells were cultured in 40 ml SC‐ura or SC‐ura + 6 mmol l^−1^ vanillin at 30°C with shaking (200 rpm) starting with an initial OD_600_ of 0.2. Data are the mean values of triplicate tests. Symbols: 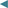, BY4741(pJFE3); 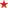, BY4741(*mbf1Δ + pJFE3*); ■, BY4741(*mbf1Δ + MBF1^WT^*); 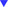, BY4741(*mbf1Δ + MBF1^P148L^*); 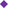, BY4741(*mbf1Δ + MBF1^D112A^*); 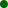, BY4741(*mbf1Δ + MBF1^P148L/D112A^*).

To investigate the effects of genes related to DNA repair (*MBF1*, *MSN1* and *WTM2*) and their mutant forms on vanillin tolerance in *S. cerevisiae*, we cloned and overexpressed these genes as we did previously for *GCY1*. We then evaluated vanillin tolerance in the resulting strains. The results (Fig. 7 and Table 2) showed that the growth characteristics of strains BY4741(*mbf1Δ + MBF1^WT^*) and BY4741(*mbf1Δ + MBF1^P148L^*) were similar to the control BY4741(pJFE3) in vanillin‐free SC‐ura medium. However, the μ of strains BY4741(*mbf1Δ + MBF1^WT^*) and BY4741(*mbf1Δ + MBF1^P148L^*) showed no differences, but were 48% (*P*‐value < 0.01) and 44% (*P*‐value < 0.01) higher, respectively, than BY4741(pJFE3) when 6 mmol l^−1^ vanillin was present in the medium. Also, the *r*_vanillin_ of these three strains were similar. The higher volumetric vanillin reduction rates of BY4741(*mbf1Δ + MBF1^WT^*) and BY4741(*mbf1Δ + MBF1^P148L^*) compared to the BY4741(pJFE3) control were due to the increases in cell biomass. These results show that overexpression of *MBF1* improves vanillin tolerance in the BY4741 strain, and the point mutation P148L did not further improve vanillin tolerance. Furthermore, the *MSN1* and *WTM2* genes and their mutants did not show any positive effects on vanillin tolerance in *S. cerevisiae* (Fig. S1 and Table S1).

### Mbf1p enhances vanillin tolerance independently of its TBP binding activity

Mbf1p (Multiprotein bridging factor 1) is an evolutionarily conserved transcriptional coactivator that has been widely studied in various organisms. Mbf1p enables interactions between the TATA element‐binding protein (TBP) and regulatory factors (Takemaru *et al*., 1997; Takemaru *et al*., 1998). In yeast, a clear example is that Mbf1p recruits TBP to promoters where the basic leucine zipper protein Gcn4p is bound to mediate GCN4‐dependent transcriptional activation of *HIS3*. It was reported that substitution of alanine for aspartate‐112 (D112A), the site where Mbf1p interacts with TBP, resulted in a weakened binding of Mbf1p and TBP and a concomitant reduction in the level of *HIS3* mRNA (Takemaru *et al*., 1998). Therefore, we introduced the point mutation D112A into *MBF1^WT^* and *MBF1^P148L^* to disrupt the binding of Mbf1p and TBP in order to study whether Mbf1p functions as a transcriptional coactivator to regulate genes that positively affect vanillin tolerance. The results (Fig. 7 and Table 2) showed that the μ of BY4741(*mbf1Δ + MBF1^D112A^*) and BY4741(*mbf1Δ + MBF1^P148L/D112A^*) were 51% (*P*‐value < 0.01) and 49% (*P*‐value < 0.01) higher than in BY4741(pJFE3), and were similar to the μ of strains overexpressing *MBF1* in the presence of 6 mM vanillin. This suggests that the weakened binding of Mbf1p and TBP did not affect the positive effect of Mbf1p on vanillin tolerance; in other words, Mbf1 enhances vanillin tolerance independent of its TBP binding activity.

## Conclusion

We identified several genes related to vanillin tolerance in *Saccharomyces cerevisiae*. Specifically, the proteins encoded by the genes *GCY1* and *YPR1* have vanillin reductase activity, so that their overexpression enhances vanillin tolerance by converting vanillin to less toxic vanillyl alcohol. However, overexpression of Gcy1p^Y56F^, the vanillin reductase‐inactive mutant allele, also increases vanillin tolerance in *S. cerevisiae*. Although the Gcy1p has RNA binding activity, and at least two genes (*TRS85* and *PEX5*) are its targets related to vanillin tolerance, the relationship between Gcy1p RNA binding activity and vanillin tolerance is unclear because neither the mRNA levels nor the protein levels of *TRS85* and *PEX5* showed significant differences between the *GCY1*‐overexpressing strain and the control. We also showed that overexpressing *MBF1* enhanced vanillin tolerance, and this is independent of the TBP binding activity of Mbf1p. Our findings offer new engineering target genes to improve the vanillin tolerance of *S. cerevisiae* and extend our understanding of the proteins encoded by these genes.

## Experimental procedures

### Strains and plasmids

The *S. cerevisiae* strains used in this study were derived from the laboratory strain BY4741 (*MATa*, *his3Δ1 leu2Δ met5Δ ura3Δ*; EUROSCARF, Germany) by gene deletion or overexpression. The deletions were performed using the homologous recombination method as previously described (Wang *et al*., 2016). The genes *GCY1^WT^*, *MBF1^WT^*, *MSN1^WT^* and *WTM2^WT^* were amplified from genomic DNA extracted from strain NAN‐27, the genes *GCY1^A181E^*, *MBF1^P148L^*, *MSN1^S171P/P190Q^* and *WTM2^Q8L^* were amplified from EMV‐8 genomic DNA, and the genes *MRPL49*, *NAM9*, *ASA1*, *VPS71*, *TEL2*, *TRS85* and *PEX5* were amplified from BY4741 genomic DNA. Other point mutations (*GCY1^Y56F^*, *GCY1^A181E/Y56F^*, *MBF1^D112A^* and *MBF1^P148L/D112A^*) in *GCY1^WT^*, *GCY1^A181E^*, *MBF1^WT^* and *MBF1^P148L^* were introduced by site‐directed PCR mutagenesis. The above‐mentioned genes were inserted into the 2μ plasmid pJFE3 under the control of the *TEF1* promoter for overexpression (Shen *et al*., 2012). The site‐directed in situ insertions of PEX5‐6*HIS and TRS85‐6*HIS into the chromosome were carried out with gRNA‐guided Cas9 nuclease as described previously (Zhang *et al*., 2014). Here, each yeast strain is named using the pattern ‘BY4741(genotype)’ for simplicity and ease of understanding. All the plasmids and strains used in this work are given in Table S3, and all the DNA primers used in this work are given in Table S4.

### Medium and culture conditions

The *Escherichia coli* strains used for subcloning were cultured in Luria‐Bertani medium (5 g l^−1^ yeast extract, 10 g l^−1^ tryptone, 10 g l^−1^ NaCl) at 37°C, and the transformants were selected on Luria‐Bertani agar plates containing additional 100 mg l^−1^ ampicillin. The *S. cerevisiae* strain BY4741 and the gene knockout strains were cultured in YPD medium (5 g l^−1^ yeast extract, 10 g l^−1^ tryptone, supplemented with 20 g l^−1^ glucose), and the gene knockout transformants were selected on YPD plates containing 800 mg l^−1^ of the antibiotic G418. The *S. cerevisiae* strains carrying plasmids were cultured in SD medium (1.7 g l^−1^ yeast nitrogen base, 5 g l^−1^ ammonium sulphate) supplemented with CSM‐URA (uracil single dropout of complete supplement mixture, MP Biomedicals, Santa Ana, California, USA) and 20 g l^−1^ glucose.

### Spot dilution growth assay

The vanillin sensitivity of the gene knockout strains was tested by the spot dilution growth assay. Overnight cultures were transferred into fresh YPD medium with an initial OD_600_ of 0.2 and cultured for another 12 h. The cells were then harvested and washed three times with ddH_2_O and resuspended in ddH_2_O. The suspension was incubated at 30°C for 9 h and then diluted to an OD_600_ of 1.0 with ddH_2_O. Ten‐fold serial dilutions were prepared, and 4 μl aliquots of each cell suspension were spotted onto YPD or vanillin‐containing YPD plates and incubated at 30°C for 1 day (YPD) and 2‐3 days (YPD with vanillin).

### Growth assay

The cells were pre‐cultured in 50‐ml flasks containing 20 ml SC‐ura medium for 12 h at 30°C with shaking at 200 rpm, and then transferred into 20 ml fresh medium and cultured another 12 h. After that the cells were transferred into 100 ml‐flasks containing 40 ml of SC‐ura medium with or without vanillin at an initial OD_600_ of 0.2. The growth rates (μ) were estimated as the linear regression coefficients of the ln OD_600_ versus time during the exponential growth phase (Peng *et al*., 2012).

### HPLC analysis of vanillin and calculation of the specific vanillin reduction rates

The concentrations of vanillin were determined using a Prominence LC‐20A HPLC system (Shimadzu, Japan) equipped with a BioSil‐C18 column (Bio‐Rad, USA). The mobile phase was 40% aqueous methanol at a flow rate of 0.6 ml min^−1^ at 37°C. The peaks were detected using an ultraviolet detector (SPD‐M20A) at 210 nm (Wang *et al*., 2017). The specific vanillin reduction rates (*r*_vanillin_) were calculated using the following equation:

$$r=\frac{A_{n}‐A_{m}}{\frac{1}{2}\sum_{i=m+1}^{n}\left(B_{i}+B_{i‐1}\right)\times \left(t_{i}‐t_{i‐1}\right)}$$

where *r* is the specific consumption rate during the phase from sampling point *m* to sampling point *n*; *A*, *B* and *t* are vanillin concentration, biomass concentration (an OD_600_ of 1 corresponds to 0.18 g l^−1^ cell biomass for strain BY4741) and time, respectively, at sampling points *n*, *i* and *m*, as previously described (Peng *et al*., 2012).

### Enzyme activity assays

Yeast cells were cultured in SC‐ura medium and harvested when the OD_600_ reached 4.0. The collected cells were resuspended in 33 mM Na_3_PO_4_ buffer (pH = 7.0) containing 1 mM PMSF and lysed using a Precellys 24 homogenizer (Bertin Technologies, France). The homogenized cells were centrifuged at 10 000 *g* for 15 min. The supernatants were collected and used for the enzyme activity assays. The total cellular protein concentration was measured using a BCA protein assay reagent kit (Sangon Biotech, China).

The vanillin dehydrogenase activity was performed based on the method of Larroy *et al*. (2002). The activity was monitored spectrophotometrically by measuring the rate of decrease in absorption at 365 nm and was calculated using an extinction coefficient of 7.71 mM^−1^ cm^−1^ for vanillin and NAD(P)H. The reaction mixture contained, in a total volume of 0.6 ml, 33 mM sodium phosphate buffer (pH 7.0), 0.5 mM NAD(P)H, and 1 mM vanillin (using 0.2‐cm path length cuvettes). One‐unit (U) of vanillin reductase activity is defined as the amount of crude extract required to reduce 1 μmol of NAD(P)H plus vanillin per minute under the assay conditions.

### Quantification of NAD(P)^+^and NAD(P)H

Cells were cultured in a 100‐ml flask containing 40 ml SC‐ura medium. The initial OD_600_ was 0.2. Samples were prepared as previously described (Zhou *et al*., 2013). When the OD_600_ reached 4.0, cells were collected by centrifugation at 10 000 *g* for 5 min and washed twice with ice‐cold phosphate‐buffered saline (PBS). The cells were resuspended in 300 µl 0.2 mol l^−1^ NaOH (for the extraction of NAD(P)H) or 300 µl 0.2 mol l^−1^ HCl (for the extraction of NAD(P)^+^), and then repeatedly frozen in liquid nitrogen and thawed at 55°C. After the freeze‐thaw treatment, the extracts were neutralized by adding 300 µl 0.1 mol l^−1^ HCl or 300 µl of 0.1 mol l^−1^ NaOH, respectively, and centrifuged at 10 000 *g* for 15 min. The supernatants were then collected for the assays described below. The total cellular protein concentration was measured using a BCA protein assay reagent kit (Sangon Biotech, China).

The concentration of NADP(H) was quantified as previously described (Zhou *et al*., 2011; Zhou *et al*., 2013). The reaction mixture contained 0.1 mol l^−1^ Bicine buffer (pH 8.0), 3 mmol l^−1^ glucose‐6‐phosphate, 4 mmol l^−1^ EDTA (pH 8.0), 0.42 mmol l^−1^ 3‐(4,5‐dimethyl‐2‐thiazolyl)‐2,5‐diphenyl‐2H‐tetrazolium bromide, 1.66 mmol l^−1^ phenazine ethosulfate, 50 µl neutralized extract, and 50 µl of yeast glucose‐6‐phosphate dehydrogenase (10 U ml^−1^, Sigma, USA) in a total volume of 1 ml. The NADP(H) assay started immediately when the neutralized extract and glucose‐6‐phosphate dehydrogenase were added, and the absorbance at 570 nm was recorded for 2 min. For NAD(H) determination, a final concentration of 10% (v/v) ethanol was used instead of glucose‐6‐phosphate in the reaction mixture, and the reaction was started by adding 50 µl of yeast ethanol dehydrogenase II (500 U ml^−1^ in Bicine buffer; Sigma, USA) instead of glucose‐6‐phosphate dehydrogenase. The standard curves of NAD(P)H and NAD(P)^+^ were constructed using a gradient of NAD(P)H or NA(D)P^+^ standard concentrations instead of the neutralized extract.

### His‐tag ELISA detection

The levels of Trs85p and Pex5p in strains were expressed as (the amount of Trs85p or Pex5p)/(the total amount of protein). The total amount of protein in the cell‐free extracts was determined with a BCA protein assay reagent kit (Sangon Biotech, China) as described above. The amounts of Pex5p and Trs85p in cell‐free total protein extracts were determined using the His‐tag ELISA Detection Kit (Genscript, USA), which is based on competitive ELISA, according to the manufacturer's instructions. Briefly, the samples (in this study, the His‐tagged Pex5p or Trs85p) and the His‐tagged protein which is pre‐coated on the microwell plate compete to bind the anti‐His tag mAb, which can react with the HRP‐conjugated antibody. The TMB substrate then undergoes catalysis by HRP and produces a visible colour change that can be measured in a spectrophotometer. The standard curves were constructed using a gradient of His‐protein standard concentrations.

## Conflict of interest

The authors declare no conflict of interest.

## Supporting information


**Fig. S1.** Growth and vanillin reduction curves of recombinant *S. cerevisiae* strains with different *MSN1* and *WTM1* alleles in medium with or without vanillin.
**Table S1.** Growth and vanillin reduction parameters of recombinant *S. cerevisiae* strains with *MSN1* and *WTM1* alleles in medium with or without vanillin.
**Table S2.** Genes with PTS1, the C‐terminal tripeptide signal sequence, in *S. cerevisiae*.
**Table S3.** The plasmids and yeast strains used in this study.
**Table S4.** Oligonucleotide primers used in this study.
