# Supplementary material for: Newly identified genes contribute to vanillin tolerance in Saccharomyces cerevisiae
Source: Microb Biotechnol. 2020 Jul 30;14(2):503–16. doi: 10.1111/1751-7915.13643 (PMC7936312; doi:10.1111/1751-7915.13643)
Supplement: Supplementary file 1 — Fig. S1. Growth and vanillin reduction curves of recombinant S. cerevisiae strains with different MSN1 and WTM1 alleles in medium with or without vanillin. Table S1. Growth and vanillin reduction parameters of recombinant S. cerevisiae strains with MSN1 and WTM1 alleles in medium with or without vanillin. Table S2. Genes with PTS1, the C‐terminal tripeptide signal sequence, in S. cerevisiae. Table S3. The plasmids and yeast strains used in this study. Table S4. Oligonucleotide primers used in this study. [file MBT2-14-503-s001.docx]

**Fig. S1 Growth and vanillin reduction curves of recombinant *S. cerevisiae* strains with different *MSN1* and *WTM1* alleles in medium with or without vanillin.** (A and D), growth curve in SC-ura medium; (B and E), growth curve in SC-ura with additional 6 mmol L^-1^ vanillin; (C and F), vanillin reduction in SC-ura with additional 6 mmol L^-1^ vanillin. The cells were cultured in 40 mL SC-ura or SC-ura with 6 mmol L^-1^ vanillin at 30 °C, 200 rpm with an initial OD_600_ of 0.2. Data are the mean values of triplicate tests. Symbols:◄, BY4741(pJFE3); ■, BY4741(*msn1Δ+MSN1^WT^*); ●, BY4741(*msn1Δ+pJFE3*); ▲, BY4741(*msn1Δ+MSN1^S171P/P190Q^*); ★, BY4741(*wtm2Δ+pJFE3*); ▼, BY4741(*wtm2Δ+WTM2^WT^*); ◆, BY4741(*wtm2Δ+WTM2^Q8L^*)*.*

**Table S1 Growth and vanillin reduction parameters of recombinant *S. cerevisiae* strains with *MSN1* and *WTM1* alleles in medium with or without vanillin.**

| **Strains** | **SC-ura medium** | **SC-ura medium with**  **6 mmol L^-1^ vanillin** | |
| --- | --- | --- | --- |
|  | **µ**  **(h^−1^)** | **µ**  **(h^−1^)** | **r_vanillin_**  **(g L^−1^ h^−1^ g^−1^ DCW)** |
| BY4741(pJFE3) | 0.343±0.003 | 0.061±0.001 | 0.044±0.002 |
| BY4741(*msn1Δ+pJFE3*) | 0.348±0.001 | 0.049±0.002 | 0.048±0.001 |
| BY4741(*msn1Δ+MSN1^WT^*) | 0.386±0.001 | 0.040±0.007 | 0.047±0.000 |
| BY4741(*msn1Δ+MSN1^S171P/P190Q^*) | 0.362±0.005 | 0.049±0.004 | 0.046±0.002 |
| BY4741(*wtm2Δ+pJFE3*) | 0.377±0.003 | 0.049±0.000 | 0.044±0.001 |
| BY4741(*wtm2Δ+WTM2^WT^*) | 0.286±0.000 | 0.044±0.000 | 0.044±0.002 |
| BY4741(*wtm2Δ+WTM2^Q8L^*) | 0.304±0.001 | 0.045±0.004 | 0.045±0.003 |

μ, the specific growth rates; r_vanillin_, the speciﬁc vanillin reduction rate; DCW, dry cell weight. The parameters were calculated basing on the data of exponential growth phase, and are given as the averages ± standard deviation of the biological triplicates.

**Table S2 Genes with PTS1, the C-terminal tripeptide signal sequence, in *S. cerevisiae.***

| **ORF Name** | **Gene Name** | **Description** |
| --- | --- | --- |
| YLR027C | AAT2 | Cytosolic aspartate aminotransferase, involved in nitrogen metabolism; localizes to peroxisomes in oleate-grown cells |
| YLR109W | AHP1 | Thiol-specific peroxiredoxin, reduces hydroperoxides to protect against oxidative damage; function in vivo requires covalent conjugation to Urm1p |
| YML042W | CAT2 | Carnitine acetyl-CoA transferase present in both mitochondria and peroxisomes, transfers activated acetyl groups to carnitine to form acetylcarnitine which can be shuttled across membranes |
| YCR005C | CIT2 | Citrate synthase, catalyzes the condensation of acetyl coenzyme A and oxaloacetate to form citrate, peroxisomal isozyme involved in glyoxylate cycle; expression is controlled by Rtg1p and Rtg2p transcription factors |
| YOR180C | DCI1 | Peroxisomal protein; identification as a delta(3,5)-delta(2,4)-dienoyl-CoA isomerase involved in fatty acid metabolism is disputed |
| YLR284C | ECI1 | Peroxisomal delta3,delta2-enoyl-CoA isomerase, hexameric protein that converts 3-hexenoyl-CoA to trans-2-hexenoyl-CoA, essential for the beta-oxidation of unsaturated fatty acids, oleate-induced |
| YKR009C | FOX2 | Multifunctional enzyme of the peroxisomal fatty acid beta-oxidation pathway; has 3-hydroxyacyl-CoA dehydrogenase and enoyl-CoA hydratase activities |
| YGR154C | GTO1 | Omega-class glutathione transferase; induced under oxidative stress; putative peroxisomal localization |
| YDR234W | LYS4 | Homoaconitase, catalyzes the conversion of homocitrate to homoisocitrate, which is a step in the lysine biosynthesis pathway |
| YNL009W | IDP3 | Peroxisomal NADP-dependent isocitrate dehydrogenase, catalyzes oxidation of isocitrate to alpha-ketoglutarate with the formation of NADP(H+), required for growth on unsaturated fatty acids |
| YGR077C | PEX8 | Intraperoxisomal organizer of the peroxisomal import machinery, tightly associated with the lumenal face of the peroxisomal membrane, essential for peroxisome biogenesis, binds PTS1-signal receptor Pex5p |
| YOR084W | LPX1 | Oleic acid-inducible, peroxisomal matrix localized lipase; transcriptionally activated by Yrm1p along with genes involved in multidrug resistance; peroxisomal import is dependent on the PTS1 receptor, Pex5p and on self-interaction |
| YIR034C | LYS1 | Saccharopine dehydrogenase (NAD+, L-lysine-forming), catalyzes the conversion of saccharopine to L-lysine, which is the final step in the lysine biosynthesis pathway; also has mRNA binding activity |
| YNL117W | MLS1 | Malate synthase, enzyme of the glyoxylate cycle, involved in utilization of non-fermentable carbon sources; expression is subject to carbon catabolite repression; localizes in peroxisomes during growth in oleic acid medium |
| YDL078C | MDH3 | Peroxisomal malate dehydrogenase, catalyzes interconversion of malate and oxaloacetate; involved in the glyoxylate cycle |
| YGL067W | NPY1 | NADH diphosphatase (pyrophosphatase), hydrolyzes the pyrophosphate linkage in NADH and related nucleotides; localizes to peroxisomes |
| YBR222C | PCS60 | Peroxisomal protein that binds AMP and mRNA, localizes to both the peroxisomal peripheral membrane and matrix, expression is highly inducible by oleic acid, similar to *E. coli* long chain acyl-CoA synthetase |
| YNL202W | SPS19 | Peroxisomal 2,4-dienoyl-CoA reductase, auxiliary enzyme of fatty acid beta-oxidation; homodimeric enzyme required for growth and sporulation on petroselineate medium; expression induced during late sporulation and in the presence of oleate |
| YJR019C | TES1 | Peroxisomal acyl-CoA thioesterase likely to be involved in fatty acid oxidation rather than fatty acid synthesis; conserved protein also found in human peroxisomes; TES1 mRNA levels increase during growth on fatty acids |

The genes with PTS1 was get from http://www.peroxisomedb.org/; the gene description was get from http://www.yeastract.com/. Both websites were accessed in 19 Feb, 2020.

**Table S3 The plasmids and yeast strains used in this study.**

| **Plasmids and strains** | **Genotype** | **Source/Reference** |
| --- | --- | --- |
| **Plasmids** | | |
| pUG6 | *E. coli* plasmid with segment LoxP-KanMX4-LoxP | (Guldener et al., 1996) |
| pSH47 | shuttle plasmid for *E. coli* and *S. cerevisiae*, *Cre* gene under *GAL2* regulative promoter | (Guldener et al., 1996) |
| pJFE3 | 2 μ expression vector with URA3 marker, *TEF1* promoter, *PGK1* terminator | (Shen et al., 2012) |
| Cas9-NAT | Cas9 expression plasmid | (Zhang et al., 2014) |
| gRNA-trp-HYB | Customized gRNA-expressing plasmid | (Zhang et al., 2014) |
| gRNA-trs85-HYB | *TRS85* disruption gRNA cassette | this work |
| gRNA-pex5-HYB | *PEX5* disruption gRNA cassette | this work |
| pJFE3-GCY1^WT^ | *GCY1* ORF segments (amplificated from the genomic DNA of NAN-27) cloned into pJFE3 | this work |
| pJFE3-GCY1^A181E^ | *GCY1* ORF segments (amplificated from the genomic DNA of EMV-8) cloned into pJFE3 | this work |
| pJFE3-GCY1^Y56F^ | *GCY1* ORF segments (amplificated from the genomic DNA of NAN-27 with mutation Y56F) cloned into pJFE3 | this work |
| pJFE3-GCY1^A181E/Y56F^ | *GCY1* ORF segments (amplificated from the genomic DNA of EMV-8 with mutation Y56F) cloned into pJFE3 | this work |
| pJFE3-YPR1 | *YPR1* ORF segments (amplificated from the genomic DNA of BY4741) cloned into pJFE3 | this work |
| pJFE3-MBF1^WT^ | *MBF1* ORF segments (amplificated from the genomic DNA of NAN-27) cloned into pJFE3 | this work |
| pJFE3-MBF1^P148L^ | *MBF1* ORF segments (amplificated from the genomic DNA of EMV-8) cloned into pJFE3 | this work |
| pJFE3-MBF1^D112A^ | *MBF1* ORF segments (amplificated from the genomic DNA of NAN-27 with mutation Y56F) cloned into pJFE3 | this work |
| pJFE3-MBF1^P148L/D112A^ | *MBF1* ORF segments (amplificated from the genomic DNA of EMV-8 with mutation Y56F) cloned into pJFE3 | this work |
| pJFE3-MSN1^WT^ | *MSN1* ORF segments (amplificated from the genomic DNA of NAN-27) cloned into pJFE3 | this work |
| pJFE3-MSN1^S171P/P190Q^ | *MSN1* ORF segments (amplificated from the genomic DNA of EMV-8) cloned into pJFE3 | this work |
| pJFE3-WTM2^WT^ | *WTM2* ORF segments (amplificated from the genomic DNA of NAN-27) cloned into pJFE3 | this work |
| pJFE3-WTM2^Q8L^ | *WTM2* ORF segments (amplificated from the genomic DNA of EMV-8) cloned into pJFE3 | this work |
| pJFE3-MRPL49 | *MPRL49* ORF segments (amplificated from the genomic DNA of BY4741) cloned into pJFE3 | this work |
| pJFE3-NAM9 | *NAM9* ORF segments (amplificated from the genomic DNA of BY4741) cloned into pJFE3 | this work |
| pJFE3-ASA1 | *ASA1* ORF segments (amplificated from the genomic DNA of BY4741) cloned into pJFE3 | this work |
| pJFE3-TEL2 | *TEL2* ORF segments (amplificated from the genomic DNA of BY4741) cloned into pJFE3 | this work |
| pJFE3-VPS71 | *VPS71* ORF segments (amplificated from the genomic DNA of BY4741) cloned into pJFE3 | this work |
| pJFE3-TRS85 | *TRS85* ORF segments (amplificated from the genomic DNA of BY4741) cloned into pJFE3 | this work |
| pJFE3-PEX5 | *PEX5* ORF segments (amplificated from the genomic DNA of BY4741) cloned into pJFE3 | this work |
| ***S.cerevisiae* strains** | | |
| NAN-27 | 2n prototroph | (Wang et al., 2017) |
| EMV-8 | NAN-27 derivate; evolutionary in Lignocellulosic hydrolysate | (Wang et al., 2017) |
| BY4741 | *MATa*; *his3Δ1*; *leu2Δ*; *met5Δ*; *ura3Δ* | EUROSCARF |
| BY4741(*gcy1Δ*) | BY4741 derivate; *gcy1::loxP-KanMX4-loxP* | this work |
| BY4741(*gut2Δ*) | BY4741 derivate; *gut2::loxP-KanMX4-loxP* | this work |
| BY4741(*ccs1Δ*) | BY4741 derivate; *ccs1::loxP-KanMX4-loxP* | this work |
| BY4741(*ecm5Δ*) | BY4741 derivate; *ecm5::loxP-KanMX4-loxP* | this work |
| BY4741(*sfl1Δ*) | BY4741 derivate; *sfl1::loxP-KanMX4-loxP* | this work |
| BY4741(*ubp2Δ*) | BY4741 derivate; *ubp2::loxP-KanMX4-loxP* | this work |
| BY4741(*ybp1Δ*) | BY4741 derivate; *ybp1::loxP-KanMX4-loxP* | this work |
| BY4741(*mbf1Δ*) | BY4741 derivate; *mbf1::loxP-KanMX4-loxP* | this work |
| BY4741(*nfi1Δ*) | BY4741 derivate; *nfi1::loxP-KanMX4-loxP* | this work |
| BY4741(*msn1Δ*) | BY4741 derivate; *msn1::loxP-KanMX4-loxP* | this work |
| BY4741(*wtm1Δ*) | BY4741 derivate; *wtm1::loxP-KanMX4-loxP* | this work |
| BY4741(*wtm2Δ*) | BY4741 derivate; *wtm2::loxP-KanMX4-loxP* | this work |
| BY4741(*rad9Δ*) | BY4741 derivate; *rad9::loxP-KanMX4-loxP* | this work |
| BY4741(*rsf2Δ*) | BY4741 derivate; *rsf2::loxP-KanMX4-loxP* | this work |
| BY4741(*pms1Δ*) | BY4741 derivate; *pms1::loxP-KanMX4-loxP* | this work |
| BY4741(pJFE3) | BY4741 derivate; pJFE3 | this work |
| BY4741(*gcy1Δ+pJFE3*) | BY4741 derivate; *gcy1::loxP-KanMX4-loxP*; pJFE3 | this work |
| BY4741(*gcy1Δ+GCY1^WT^*) | BY4741 derivate; *gcy1::loxP-KanMX4-loxP*; pJFE3-GCY1^WT^ | this work |
| BY4741(*gcy1Δ+GCY1^A181E^*) | BY4741 derivate; *gcy1::loxP-KanMX4-loxP*; pJFE3-GCY1^A181E^ | this work |
| BY4741(*gcy1Δ+GCY1^Y56F^*) | BY4741 derivate; *gcy1::loxP-KanMX4-loxP*; pJFE3-GCY1^Y56F^ | this work |
| BY4741(*gcy1Δ+GCY1^A181E/Y56F^*) | BY4741 derivate; *gcy1::loxP-KanMX4-loxP*; pJFE3-GCY1^A181E/Y56F^ | this work |
| BY4741(*YPR1*) | BY4741 derivate; pJFE3-YPR1 | this work |
| BY4741(MRPL49) | BY4741 derivate; pJFE3-MRPL49 | this work |
| BY4741(NAM9) | BY4741 derivate; pJFE3-NAM9 | this work |
| BY4741(ASA1) | BY4741 derivate; pJFE3-ASA1 | this work |
| BY4741(TEL2) | BY4741 derivate; pJFE3-TEL2 | this work |
| BY4741(VPS71) | BY4741 derivate; pJFE3-VPS71 | this work |
| BY4741(PEX5) | BY4741 derivate; pJFE3-PEX5 | this work |
| BY4741(TRS85) | BY4741 derivate; pJFE3-TRS85 | this work |
| BY4741(pJFE3)+*PEX5-6*His*(in situ) | BY4741 derivate; pJFE3, *pex5::PEX5-6*His* | this work |
| BY4741(*gcy1Δ+pJFE3*)+*PEX5-6*His*(in situ) | BY4741 derivate; *gcy1::loxP-KanMX4-loxP*; pJFE3, *pex5::PEX5-6*His* | this work |
| BY4741(*gcy1Δ+GCY1^WT^*)+*PEX5-6**His(in situ) | BY4741 derivate; *gcy1::loxP-KanMX4-loxP*; pJFE3-GCY1^WT^, *pex5::PEX5-6*His* | this work |
| BY4741(*gcy1Δ+GCY1^A181E^*)+*PEX5-6**His(in situ) | BY4741 derivate; *gcy1::loxP-KanMX4-loxP*; pJFE3-GCY1^A181E^, *pex5::PEX5-6*His* | this work |
| BY4741(pJFE3)+*TRS85-6*His*(in situ) | BY4741 derivate; pJFE3, *trs85::TRS85-6*His* | this work |
| BY4741(*gcy1Δ+pJFE3*)+*TRS85-6*His*(in situ) | BY4741 derivate; *gcy1::loxP-KanMX4-loxP*; pJFE3, *trs85::TRS85-6*His* | this work |
| BY4741(*gcy1Δ+GCY1^WT^*)+*TRS85-6*His*(in situ) | BY4741 derivate; *gcy1::loxP-KanMX4-loxP*; pJFE3-GCY1^WT^, *trs85::TRS85-6*His* | this work |
| BY4741(*gcy1Δ+GCY1^A181E^*)+*TRS85-6*His*(in situ) | BY4741 derivate; *gcy1::loxP-KanMX4-loxP*; pJFE3-GCY1^A181E^, *trs85::TRS85-6*His* | this work |
| BY4741(*mbf1Δ+pJFE3*) | BY4741 derivate; *mbf1::loxP-KanMX4-loxP;* pJFE3 | this work |
| BY4741(*mbf1Δ+MBF1^WT^*) | BY4741 derivate; *mbf1::loxP-KanMX4-loxP;* pJFE3-MBF1^WT^ | this work |
| BY4741(*mbf1Δ+MBF1^P148L^*) | BY4741 derivate; *mbf1::loxP-KanMX4-loxP;* pJFE3-MBF1^P148L^ | this work |
| BY4741(*mbf1Δ+MBF1^D112A^*) | BY4741 derivate; *mbf1::loxP-KanMX4-loxP;* pJFE3-MBF1^D112A^ | this work |
| BY4741(*mbf1Δ+MBF1^P148L/D112A^*) | BY4741 derivate; *mbf1::loxP-KanMX4-loxP;* pJFE3-MBF1^P148L/D112A^ | this work |
| BY4741(*msn1Δ+pJFE3*) | BY4741 derivate; *msn1::loxP-KanMX4-loxP;* pJFE3 | this work |
| BY4741(*msn1Δ+MSN1^WT^*) | BY4741 derivate; *msn1::loxP-KanMX4-loxP;* pJFE3-MSN1^WT^ | this work |
| BY4741(*msn1Δ+MSN1^S171P/P190Q^*) | BY4741 derivate; *msn1::loxP-KanMX4-loxP;* pJFE3-MSN1^S171P/P190Q^ | this work |
| BY4741(*wtm2Δ+pJFE3*) | BY4741 derivate; *wtm2::loxP-KanMX4-loxP;* pJFE3 | this work |
| BY4741(*wtm2Δ+WTM2^WT^*) | BY4741 derivate; *wtm2::loxP-KanMX4-loxP;* pJFE3-WTM2^WT^ | this work |
| BY4741(*wtm2Δ+WTM2^Q8L^*) | BY4741 derivate; *wtm2::loxP-KanMX4-loxP;* pJFE3-WTM2^Q8L^ | this work |

**References**

Guldener, U., Heck, S., Fielder, T., Beinhauer, J., and Hegemann, J.H. (1996). A new efficient gene disruption cassette for repeated use in budding yeast. *Nucleic Acids Res* 24(13)**,** 2519-2524. doi: 10.1093/nar/24.13.2519.

Shen, Y., Chen, X., Peng, B., Chen, L., Hou, J., and Bao, X. (2012). An efficient xylose-fermenting recombinant Saccharomyces cerevisiae strain obtained through adaptive evolution and its global transcription profile. *Appl Microbiol Biotechnol* 96(4)**,** 1079-1091. doi: 10.1007/s00253-012-4418-0.

Wang, X., Liang, Z., Hou, J., Shen, Y., and Bao, X. (2017). The absence of the transcription factor Yrr1p, identified from comparative genome profiling, increased vanillin tolerance due to enhancements of ABC transporters expressing, rRNA processing and ribosome biogenesis in Saccharomyces cerevisiae. *Front Microbiol* 8**,** 367. doi: 10.3389/fmicb.2017.00367.

Zhang, G.C., Kong, II, Kim, H., Liu, J.J., Cate, J.H., and Jin, Y.S. (2014). Construction of a quadruple auxotrophic mutant of an industrial polyploid saccharomyces cerevisiae strain by using RNA-guided Cas9 nuclease. *Appl Environ Microbiol* 80(24)**,** 7694-7701. doi: 10.1128/AEM.02310-14.

**Table S4 Oligonucleotide primers used in this study.**

| **Name** | **Primes sequence** |
| --- | --- |
| **Primers for Plasmid Construction (Restriction Sites/ Homologous with Flanking Sequence of Plasmid pJFE3)** | |
| GCY1-up | CGTACGGATCCATGCCTGCTACTTTACATGATTCTACG |
| GCY1-down | CGTACGCGTCGACTTACTTGAATACTTCGAAAGGAGACCAATTTGG |
| GCY1-Y56F-up | CTGCTGCTATTTTTCGTAATGAAGACCAAGTCGG |
| GCY1-Y56F-down | CCGACTTGGTCTTCATTACGAAAAATAGCAGCAG |
| YPR1-up | TCTAGTCTAGAATGCCTGCTACGTTAAAGAATTCTTCT |
| YPR1-down | CGTACCCTGCAGGTCATTGGAAAATTGGGAAGGATCCC |
| MBF1-up | GACTAGTCTAGAATGTCTGACTGGGATACAAATACTATTATTGGTAG |
| MBF1-edown | CGTACACCTGCAGGTCATTTCTTCTTTAGAGCTCCCAAAGGC |
| MBF1-ndown | CGTACACCTGCAGGTCATTTCTTCTTTGGAGCTCCCAAAGG |
| MBF1-D112A-up | CCGTGGTAAACGCCTATGAAGCCGCTAGAGC |
| MBF1-D112A-down | AGCTCTAGCGGCTTCATAGGCGTTTACCACGGT |
| MSN1-up | CGTACGGATCCATGGCAAGTAACCAGCACATAGGAG |
| MSN1-down | CGTACGCGTCGACTCACTTCAAAGTCTCTGGAATATGAGAACTG |
| WTM2-up | CTAAGTTTTAATTACAAAGGATCCTCTAGAATGGCGAAAAGCAAATCCAACCTG |
| WTM2-down | TCAATTCAATTCAATCCTGCAGGTCGACTCAATCGTCGTAACCTCTGCCAATG |
| NAM9-up | CCTAGTCTAGATGCCAAGAAAGGCTAATTTGCT |
| NAM9-down | CGTACACCTGCAGGTCATTTACCATTTCTCAAGTAATACATATATGCACG |
| MRPL49-up | CGTACGGATCCATGCTACAACTGAAATTCATATGGCCA |
| MRPL49-down | CGTACGCGTCGACTCAGTTCATACTCAGTTCTGATATTCTTAAGATTGT |
| VPS71-up | CCTAGTCTAGATGAAGGCGCTAGTTGAAGAGAT |
| VPS71-down | CGTACACCTGCAGGCTATCTATTTCTGCACCTAGTTTCATTATGCA |
| TEL2-up | CGTACGGATCCATGGTTTTAGAAACGCTGAAACAAGG |
| TEL2-down | CGTACGCGTCGACCTAACCTTTATTGAGAGAAATTCCTTCCTCT |
| ASA1-up | CGTACGGATCCATGAGAGGCTTTAGTAATGAGATAATCCTG |
| ASA1-down | CGTACGCGTCGACTTATATTTTATTGAGCTTTATGGTACCATCTTCATATCC |
| PEX5-up | CGTACGGATCCATGGACGTAGGAAGTTGCTCAGT |
| PEX5-down | CGTACGCGTCGACTCAAAACGAAAATTCTCCTTTAAATCTTTTCAGG |
| TRS85-up | CGTACGGATCCATGGTTTTTTCTTATGAGCACTATATGAATCTCC |
| TRS85-down | CGTACGCGTCGACTCATCCAATAAAGCTAACACTTGTTCTTGT |
| pJFE3-up | CGGTCTTCAATTTCTCAAGT |
| pJFE3-down | AGCGTAAAGGATGGGGAAAG |
| **Primers for Gene Deletion (Homologous with Flanking Sequence of *loxP-KanMX4-loxP*)** | |
| GCY1-F | CAAGGAAGGGCGGTCTTTTCTC |
| GCY1-Kup | GGTGATATCAGATCCACTAGTGGCCTATGGCGTGTTTCTCGTATGATTGTAATATGTAG |
| GCY1-Kdown | GTCGACCTGCAGCGTACGAAGCTTCAGCTGGAGAGCTGTCCAAATTTTAGCTTGC |
| GCY1-R | GCAGGTAAAGTTTTCTTGCCTTATACAC |
| GUT2-F | ATGTTTTCGGTAACGAGAAGAAGAGCTG |
| GUT2-Kup | GAAGTTATTAGGTGATATCAGATCCACTAGTGGCCTATGCTGCCTTTGTCCTTAGCCGAC |
| GUT2-Kdown | ATTAAGGGTTGTCGACCTGCAGCGTACGAAGCTTCAGCTGGCAGCATCTAGGGCACATCCT |
| GUT2-R | TTAGACACCAAACGTCTTGATGAAGTTCAC |
| ECM5-F | ATGAGTGGGCATGATTCTGTTACAAAAATAT |
| ECM5-Kup | GAAGTTATTAGGTGATATCAGATCCACTAGTGGCCTATGCTTTGACGTTTTTGCGTTTGCT |
| ECM5-Kdown | ATTAAGGGTTGTCGACCTGCAGCGTACGAAGCTTCAGCTGGTGCTGCACCACGAGATT |
| ECM5-R | TCAATTTGGAATTCCTGTGATTATGATTCTTCCAT |
| UBP2-F | ATGCCGAACGAAGATAATGAACTTCAAAAAG |
| UBP2-Kup | GAAGTTATTAGGTGATATCAGATCCACTAGTGGCCTATGGCGAGGCCAGTTATGGTCAC |
| UBP2-Kdown | ATTAAGGGTTGTCGACCTGCAGCGTACGAAGCTTCAGCTGCTTCAGTGGTAGGTTGGTGGC |
| UBP2-R | CTACTTTAGAATTCTTTTCAATGGCTCAATATCACCTTCTT |
| YBP1-F | ATGGAACCAATTGATGACTTACTTTTTGAGGT |
| YBP1-Kup | GAAGTTATTAGGTGATATCAGATCCACTAGTGGCCTATGCGGCAGATGCCAAGAAGAGT |
| YBP1-Kdown | ATTAAGGGTTGTCGACCTGCAGCGTACGAAGCTTCAGCTGCCTTAGGCAGATCCCAACCG |
| YBP1-R | TCATTTTATACCAGTAAAATAGTCATTTAGAGATTCGTTTTTATCCA |
| SFL1-F | ATGAGTGAAGAGGAAACGGTCTCAG |
| SFL1-Kup | GAAGTTATTAGGTGATATCAGATCCACTAGTGGCCTATGGTCGAGCACAGCTCAAGCCT |
| SFL1-Kdown | ATTAAGGGTTGTCGACCTGCAGCGTACGAAGCTTCAGCTGCGTGTAGCGATTCGTCCTCC |
| SFL1-R | TCACATCTTAACCTTTTTGAGTGCCGG |
| CCS1-F | ATGACCACGAACGATACATACGAGGC |
| CCS1-Kup | GAAGTTATTAGGTGATATCAGATCCACTAGTGGCCTATGAACTTATCGGCCGCAGCTT |
| CCS1-Kdown | ATTAAGGGTTGTCGACCTGCAGCGTACGAAGCTTCAGCTGCGCCTCTTATGATGGCGTC |
| CCS1-R | CTATTTGATGTTGTTGGCCAAGGCAT |
| NFI1-F | ATGGCAAGTGTCATGTCAAATAATAATAA |
| NFI1-Kup | GAAGTTATTAGGTGATATCAGATCCACTAGTGGCCTATGCACATCGGCGGCACATCT |
| NFI1-Kdown | ATTAAGGGTTGTCGACCTGCAGCGTACGAAGCTTCAGCTGGCCTTAGGCCTCCACGGA |
| NFI1-R | TCAATCTGATGTTAAATCGACCACTTCAG |
| MBF1-F | GACGCTATCGACACCCGCA |
| MBF1-Kup | GAAGTTATTAGGTGATATCAGATCCACTAGTGGCCTATGCCATCACAACAATGGGGTCC |
| MBF1-Kdown | ATTAAGGGTTGTCGACCTGCAGCGTACGAAGCTTCAGCTGGGGCCAAGTATCAGGCCTAC |
| MBF1-R | GGGCATGATCATACCAACGGAG |
| MSN1-F | ATGGCAAGTAACCAGCACATAGGAG |
| MSN1-Kup | GAAGTTATTAGGTGATATCAGATCCACTAGTGGCCTATGACGGGCAGCCTTCTATAAGG |
| MSN1-Kdown | ATTAAGGGTTGTCGACCTGCAGCGTACGAAGCTTCAGCTGAGCGTGGCGTTGTTGG |
| MSN1-R | TCACTTCAAAGTCTCTGGAATATGAGAACTG |
| WTM1-F | ATGCCAAAAAAGGTTTGGAAATCATCTACG |
| WTM1-Kup | GAAGTTATTAGGTGATATCAGATCCACTAGTGGCCTATGCTGTGGGCGGCACTGGT |
| MTM1-Kdown | ATTAAGGGTTGTCGACCTGCAGCGTACGAAGCTTCAGCTGCACCGGTTCTGAAGCGGC |
| WTM1-R | CTATTCGCTTTCCTCGGTATAGGGC |
| WTM2-F | ATGGCGAAAAGCAAATCCAACCTG |
| WTM2-Kup | GAAGTTATTAGGTGATATCAGATCCACTAGTGGCCTATGCGGTTGGAGGAACGGGTAAT |
| WTM2-Kdown | TATTAAGGGTTGTCGACCTGCAGCGTACGAAGCTTCAGCTGGACTTCGTCTTGCCGTTACCT |
| WTM2-R | TCAATCGTCGTAACCTCTGCCAATG |
| RAD9-F | ATGTCAGGCCAGTTAGTTCAATGG |
| RAD9-Kup | GAAGTTATTAGGTGATATCAGATCCACTAGTGGCCTATGGCTTTTGCATGTTTGAGCGC |
| RAD9-Kdown | ATTAAGGGTTGTCGACCTGCAGCGTACGAAGCTTCAGCTGGGTCAAGGTCAGGAGTCCTTTC |
| RAD9-R | TCATCTAACCTCAGAAATAGTGTTGTATATATCATTG |
| RSF2-F | ATGTTTGTGAACGGTAATCAATCTAATTTCGC |
| RSF2-Kup | GAAGTTATTAGGTGATATCAGATCCACTAGTGGCCTATGTGGCAGGACAAGGATGCCC |
| RSF2-Kdown | ATTAAGGGTTGTCGACCTGCAGCGTACGAAGCTTCAGCTGCGTGGATCCCCCGTACCTAC |
| RSF2-R | TTATATTCTAGTGTTTCTTTTTTTCGTAACACTATAGATAGCTC |
| PMS1-F | ATGACACAAATTCATCAGATAAACGATATAGATGTTC |
| PMS1-Kup | GAAGTTATTAGGTGATATCAGATCCACTAGTGGCCTATGGACGGTGGGTTACGAAGAGAC |
| PMS1-Kdown | ATTAAGGGTTGTCGACCTGCAGCGTACGAAGCTTCAGCTGGGGCCTCCCCTCTAAACCC |
| PMS1-R | TCATATTTCGTAATCCTTCGAAAATGAGCTCC |
| loxP-up | CAGCTGAAGCTTCGTACGCTG |
| loxP-down | GCATAGGCCACTAGTGGATCTG |
| **Primers for Real-time Quantitative PCR** | |
| PEX5-RT-F | TGAAAGTGCGTTGAGGGTGA |
| PEX5-RT-R | ACCGCCAGATTATAGCGAGC |
| TRS85-RT-F | TGTTACAGAGCCAGTGCGTT |
| TRS85-RT-R | GTCCCAACTTGCACTCGTCT |
| actin-F | CAAACCGCTGCTCAATCTTC |
| actin-R | AGTTTGGTCAATACCGGCAG |
| **Primers for CRISPR** | |
| TRS85-ce-up | CCTCTTTATTCAGTCGGCTTTACAGATAC |
| TRS85-ce-down | GACACGAAGTACCAAATTTTTCTCGATATG |
| TRS85-donor-F | GGAGACAGGTTTCTTGGTGCT |
| TRS85-donor-up2 | TGTTAGCTTTATTGGACATCACCATCACCATCACTGA |
| TRS85-donor-down1 | TCAGTGATGGTGATGGTGATGTCCAATAAAGCTAACA |
| TRS85-donor-R | GAAGGGTCCTTTAGTAGATAAACAAAGGCA |
| TRS85gRNA-UP | AGCTTTATTGGATGACTTTAGTTTTAGAGCTAGAAATAGCAAGTTAAAATAAGGC |
| TRS85gRNA-down | TAAAGTCATCCAATAAAGCTGATCATTTATCTTTCACTGCGGAGAAGT |
| pex5-ce-up | CATAACACATGGACGTAGGAAGTTG |
| pex5-ce-down | GGTTGGATATAGGAAGTTGGGAATTATAAGG |
| PEX5-donor-F | GCAGGCTACTTATTAAGTGTTCTAAGTATGC |
| PEX5-donor-up2 | AGGAGAATTTTCGTTTCATCACCATCACCATCACTGA |
| PEX5-donor-down1 | TCAGTGATGGTGATGGTGATGAAACGAAAATTCTCCT |
| PEX5-donor-R | GTAAATAGAGCAGCAATTATGACCTTTGAATG |
| PEX5gRNA-UP | GGAGAATTTTCGTTTTGATAGTTTTAGAGCTAGAAATAGCAAGTTAAAATAAGGCT |
| PEX5gRNA-down | TATCAAAACGAAAATTCTCCGATCATTTATCTTTCACTGCGGAGAAGT |
